# FTO mediated ERBB2 demethylation promotes tumor progression in esophageal squamous cell carcinoma cells

**DOI:** 10.1007/s10585-022-10169-4

**Published:** 2022-05-07

**Authors:** Fangfang Zhao, Fangfang Ge, Minghua Xie, Zhenyu Li, Chunbao Zang, Lingsuo Kong, Youguang Pu, Xucai Zheng, Yiao Tan

**Affiliations:** 1 Department of Cancer Epigenetics Program, the First Affiliated Hospital of USTC, Division of Life Sciences and Medicine, University of Science and Technology of China, Anhui Provincial Cancer Hospital, Hefei, 230031 Anhui People’s Republic of China; 2 Department of Provincial Clinical College, Wannan Medical College, Wuhu, 241002 Anhui People’s Republic of China; 3 Department of Thoracic Tumor Surgery Department, the First Affiliated Hospital of USTC, Division of Life Sciences and Medicine, University of Science and Technology of China, Anhui Provincial Cancer Hospital, Hefei, 230031 Anhui People’s Republic of China; 4 Department of Radiation Oncology, the First Affiliated Hospital of USTC, Division of Life Sciences and Medicine, University of Science and Technology of China, Anhui Provincial Cancer Hospital, Hefei, 230031 Anhui People’s Republic of China; 5 Department of Anesthesiology, the First Affiliated Hospital of USTC, Division of Life Sciences and Medicine, University of Science and Technology of China, Anhui Provincial Cancer Hospital, Hefei, 230031 Anhui People’s Republic of China; 6 Department of Head, Neck and Breast Surgery, the First Affiliated Hospital of USTC, Division of Life Sciences and Medicine, University of Science and Technology of China, Anhui Provincial Cancer Hospital, Hefei, 230031 Anhui People’s Republic of China; 7 Department of Urology Surgery, the First Affiliated Hospital of USTC, Division of Life Sciences and Medicine, University of Science and Technology of China, Anhui Provincial Cancer Hospital, Hefei, 230031 Anhui People’s Republic of China

**Keywords:** Esophageal squamous cell carcinoma, N^6^-methyladenosine (m^6^A) modification, FTO, ERBB2, YTHDF1

## Abstract

N^6^-methyladenosine (m^6^A) is the most prevalent and internal modification that occurs in the messenger RNAs of eukaryotes. However, knowledge of the impact of these modifications on gene expression regulation remains limited. By using the in vitro MeRIP-seq and RNA-seq assays, we discovered that the mRNA demethylase FTO was significantly up-regulated in esophageal squamous cell carcinoma (ESCC) tissues and cells. Knockdown of FTO drastically suppressed the proliferation, migration, and invasion of ESCC cells. Furthermore, by using transcriptome-wide m^6^A-seq and RNA-seq assays, we identified ERBB2 is the target of FTO, which acts in concert in ESCC tumorigenesis and metastasis. Moreover, loss and gain functional studies suggested that the m^6^A reader YTHDF1 stabilizes ERBB2 mRNA via decoding the m^6^A modification. All these results uncovered a new signaling cascade, including FTO, YTHDF1, and ERBB2, which finely regulates the ESCC progression.

**Supplementary Information:**

The online version contains supplementary material available at 10.1007/s10585-022-10169-4.

## Introduction

Although post-transcriptional modifications are known to occur in RNAs, the biological functions of these modifications has only recently begun to be explored [1]. The mRNA m^6^A modification was first identified in the 1970s [2–5]. Since then, the accumulating studies have demonstrated this modification is most abundant in eukaryotic mRNAs with unique distribution patterns [6, 7]. In addition, m^6^A modification also exists in different types of RNAs, including ribosomal RNAs, small nuclear RNAs, and transfer RNAs [8–11]. Despite its widespread distribution in mammalian transcriptome, functional studies remain limited, possibly due to the low abundance of m^6^A mRNA and technical difficulties in global detection.

In mammalian cells, the m^6^A modification is catalyzed by a methyltransferase complex (“writers”) consisting of the proteins methyltransferase-like 3 (METTL3) and METTL14, which form a stable heterodimer complex [12, 13]. Then the mammalian splicing factor WTAP (Wilms tumor 1 associated protein) was identified as the third auxiliary factor of the core methyltransferase complex that affects cellular m^6^A methylation [12, 14]. Notably, the first RNA demethylase, fat mass and obesity-associated protein (FTO), was identified to function as an m^6^A “eraser” to selectively reverse m^6^A to adenosine in nuclear RNA, suggesting that RNA m^6^A modification is reversible [15]. Afterwards, the alkylation repair homolog protein 5 (ALKBH5) was proved to be another “eraser” of m^6^A modification [16, 17], indicating a dynamic nature of m^6^A methylation. Since then, the m^6^A-binding proteins with YTH domain, including cytoplasmic protein YTHDF1, YTHDF2, YTHDF3, and nuclear protein YTHDC1, have been identified to be the “readers” of m^6^A, which preferentially recognizes m^6^A-containing mRNA to modulate its stability and translation [7, 18–20].

Numerous studies have demonstrated that mRNA m^6^A modifications play important roles in various biological processes, such as heat-shock response [19], DNA damage response [21, 22], mRNA clearance [23], neuronal functions [24], cortical neurogenesis [25], progenitor cell specification [26], and T-cell homeostasis [27]. Moreover, the m^6^A modifications are commonly occurred in eukaryotes, which involved in cancer progression under multiple regulatory mechanisms [28]. As the first identified demethylase of mRNA m^6^A modification, FTO with single nucleotide polymorphism (SNP) is associated with cancer susceptibility and tumorigenesis [29–31]. In addition, the increasing evidences showed that overexpression of FTO in a variety of tumor tissues was highly correlated with the prognosis of tumors [32]. In vivo experiments showed that FTO promoted the invasion capability of AML cells and enhanced the cell transformation mediated by leukemia oncogenes [33]. Owing to the complicated regulatory mechanisms, the FTO targets and their definitive roles in cancer remained limited.

In this study, we investigated the role of m^6^A modification in the tumorigenesis of ESCC, and found a significant increased level of FTO in ESCC. In vitro assays showed that the epidermal growth factor receptor ERBB2 (Erb-B2 Receptor Tyrosine Kinase 2) [34] is a downstream target of FTO. Further studies demonstrated that FTO and ERBB2 act in concert to regulate the tumorigenesis and metastasis of ESCC. Our findings suggested that FTO mediated ERBB2 m^6^A modification might be applied to offer a new therapeutic strategy to treat ESCC.

## Materials and methods

### The ESCC specimens and cell lines

A total of 28 cases of ESCC and its paracancerous tissues with histologically confirmed ESCC were obtained by tumor surgical resection at Anhui Provincial Cancer Hospital, West Branch of the First Affiliated Hospital of University of Science and Technology of China (USTC). All experimental procedures were conducted by Biomedical Ethics Committee of USTC. All participants have signed informed consent, and their clinicopathological information is shown in Table S1 of the SI Appendix.

Human normal esophageal epithelial cell lines HEEC and esophageal squamous cell carcinoma (ESCC) cell lines KYSE140, KYSE180, KYSE450, KYSE30 and KYSE150 were acquired from China Cell Resource Center (Shanghai, China) and cultured in RPMI 1640 (BI) medium supplemented with 10% fetal bovine serum (PAN) at 37 °C and 5% CO_2._ All cell lines had been tested negative for mycoplasma.

### Plasmids and transfection

Stable knockdown and overexpression of FTO and ERBB2 were achieved by lentivirus which were purchased from Hanbio (Shanghai, china) and carried short-hairpin RNA (shRNA) and open reading frames of the genes (ORF). The flag labeled YTHDF1, YTHDF2, YTHDF3, YTHDC1 and YTHDC2 plasmids were constructed by pcDNA3.1-3XFlag vector. TheYTHDF1 ORF was inserted into PCDH vector to construct YTHDF1 overexpression plasmid. The PLKO.1 plasmid carrying YTHDF1 short hairpin RNA (shRNA) was constructed to knockout YTHDF1expression. Small interfering RNA (siRNA) targeting ERBB2-specific regions were synthesized by Ruibo (Guangzhou, China). Transfections were carried out using the Lipofectamine2000 (Invitrogen, USA) following the manufacturer’s instructions. The siRNAs and shRNAs sequences were listed in Table S2 of the SI Appendix.

### RNA extraction and real-time PCR

Total RNA was isolated from cell lines by TRIzol reagent (Vazyme). cDNA was synthesized using HiScript®II 1st Strand cDNA Synthesis Kit (Vazyme). The relative target gene mRNA expression was analyzed using the comparative CT method, with GAPDH as the endogenous control. All the qPCR primer sequences were shown in SI Appendix, Table S2.

### Western blot assays

ESCC tissues, paracancerous tissues, cultured cells were lysed by lysis buffer (60 mM Tris–HCl, pH 6.8, 2% SDS, 20% Glycerol, 0.25% bromophenol blue and 1.25% 2-mercaptoethanol). The proteins were separated by SDS-PAGE and transferred to PVDF membrane (Millipore). The membrane was blocked in 5% skimmed milk for 1 h and then incubated overnight with specific antibodies at 4 °C, followed by treatment of horseradish peroxidase-conjugated secondary antibody (Proteintech). The signal bands were detected with ECL kit (Thermo). All antibodies used in this paper are from Proteintech. Detailed information regarding the full-length gels is depicted in SI Appendix, Figs. S2–S11.

### Cell proliferation assays

A total of 3000 transfected cells were cultured into 96-well plates, the cells were added with CCK-8 solution (Bimake) at 0, 24, 48, 72, and 96 h after cell adherent according to the manufacturer’s instructions and incubated for additional 2 h. The absorbance values were measured at 450 nm by Universal Microplate Spectrophotometer.

### Colony formation assays

Different treated cells were resuspended and seed into each well of the six-well plate with a density of 500 cells. After 2 weeks, the clones were fixed with alcohol and then stained with crystal violet, and the number of cells was counted.

### In vitro migration and invasion assays

The treated cells in 200 μl serum-free medium (Cell density is 2.5 × 10^5^ for migration assays, and 8 × 10^4^ for invasion assays) were loaded in a 24-well transwell inserts upper compartment with an 8 μm diameter pores (Corning). Transwell chamber was covered with matrigel (BD) for invasion assay, but no matrix glue for migration assays. The medium of 600 μl containing 20% serum was added to the lower compartment. After 40 h, the non-migrating and non-invasive cells were scraped off, the migrating and invasive cells were fixed on the lower surface, stained with crystal violet solution, counted and photographed.

### Methylated RNA immunoprecipitation sequencing (MeRIP-seq) and RNA-seq

Total RNA was first lyextracted from stably knockdown FTO KYSE150 cells and their corresponding controls. Then the mRNA was isolated from the total RNA by PolyATtract® mRNA Isolation Systems (Promega). M^6^A methylated mRNA fragments were enriched using immunomagnetic beads with m^6^A antibody. The enriched mRNA fragments were purified, constructed a high-throughput sequencing library using Illumina’s Nebnext Ultra RNA library preparation kit and sequenced by Illumina HiSeq 2000. At the same time, a common transcriptome library should be constructed separately as a control. Library preparation and high-throughput sequencing were performed by Novo gene. Reads Mapping, m^6^A Peak Calling, Motif Search, and Following analysis were performed as described previously [35].

### Quantification of RNA m^6^A modification

RNA m^6^A methylation status in total RNAs was detected using the m^6^A RNA Methylation Quantitative Kit (Epigentek) according to the manufacturer’s instructions. In short, 200 ng RNA was bound to the strip wells. Then, m^6^A RNA capture antibody and detection antibody were added respectively, and the detection signal was enhanced with a signal enhancer. Finally, the absorbance value at 450 nm was recorded by the microplate reader, and the percentage of m^6^A in the total RNA was calculated.

### Dot blot assays

The mRNA was isolated from the total RNA using PolyATtract®mRNA Isolation Systems (Promega) Kit and denaturated, fixed on a positively charged nylon membrane (Beyotime), and then sealed with 5% skimmed milk. The m^6^A antibody (Active motif) was incubated overnight at 4 °C, and then the membrane was cleaned and incubated at room temperature for 2 h with rabbit secondary antibody (Proteintech). The signal on the nylon membrane is captured by an Image Reader. Finally, the nylon membrane was stained with 0.2% methylene blue to determine whether the sample loading quantity was the same. Detailed information regarding the full-length gels is depicted in SI Appendix, Fig. S12.

### RNA-binding protein immunoprecipitation (RIP) assays

RIP detection was performed using Magna RIP™ RNA-Binding Protein Immunoprecipitation Kit (Millipore) according to the manufacturer’s instructions. Briefly, the cell lysates prepared were incubated with magnetic beads-complex containing positive control antibody (Millipore), negative control antibody IgG (Millipore), and Flag antibody at 4 °C for 3 h or overnight. Then the RNA–protein complex was incubated at 55 °C for 30 min in a buffer solution with protease K. Finally, the RNA was extracted with phenol/chloroform/isoamyl alcohol. The interaction between ERBB2 and m^6^A reader proteins was identified by PCR and normalized to the input. The ERBB2 primers for PCR are detailed in the Appendix SI, Table S2.

### M^6^A-RNA immunoprecipitation(MeRIP) qPCR assay

The Magna MeRIP™ m^6^A Kit (Millipore) is used for quantitative analysis of RNA methylation levels of specific genes. Briefly, the mRNA was isolated and acquired from the total RNA using PolyA Ttract® mRNA Isolation Systems (Promega) Kit. Then, the mRNA was chemically segmented to a nucleotide fragment of about 100 bp and the mRNA containing m^6^A was enriched by immunoprecipitation reaction with m^6^A antibody. Finally, the enrichment of mRNA was detected by qPCR with m^6^A primers for ERBB2. The specific primers sequences for ERBB2 were shown in Table S2 of the SI Appendix [36, 55].

### RNA pull-down assays

Lysates from 3 × 10^7^ KYSE150 cells were collected and incubated with 4ug biotin-labeled sense or antisense ERBB2 probes. Followed by, after overnight at 4 °C of incubation, streptavidin magnetic beads were added to the reaction mix and incubated at 4 °C for 4 h. The magnetic beads were washed by pull-down buffer, added to loading buffer and boiled for western blot analysis.

### RNA stability

In order to detect the mRNA stability of knock-down FTO cells, sh-FTO and control cells were implanted into 12-well plates overnight, and then treated with Actinomycetes D (ApexBio, 6 μg/ml) for corresponding time (0, 2, 4, 6, and 8 h) to block RNA transcription. Finally, RNA was extracted with Trizol and analyzed by qPCR. The mRNA expression level of each group at the specified time was calculated and normalized with β-actin.

### Luciferase reporter assays

The wild-type or mutant PGL plasmid containing the 3′UTR fragment (in which adenosine at the m^6^A site was replaced by cytosine) of ERBB2 was transfected into the stable FTO knockdown KYSE150 with Lipofectamine 2000, respectively. Luciferase activity was detected using the dual luciferase reporting system kit (Promega) by Promega Glomax 20/20 Luminomete at 24 h after transfection, and was normalized with Renilla fluorescence. Detailed information regarding the primers used for plasmid construction is depicted in SI Appendix, Table S2.

### In vivo xenograft and metastasis model

Female BALB/c nude mice (4-week-old) was purchased from Shanghai SLAC Experimental Animal Co., Ltd. For the subcutaneous transplantation model, sh-control, sh-FTO, NC-OE and ERBB2-OE KYSE150 stable cells (6 × 10^6^ per mouse, n = 3 for each group) were diluted to 100 μl PBS + 100 μl Matrigel (BD) and subcutaneously injected to two points in the middle and upper groin of immunodeficient mice to study tumor growth. When the tumor volume reached ~ 1000 mm^3^ in each group, the nude mice were killed, and the tumors were excised and weighed for histology and further study. The tumor volume was calculated by the formula V = 1/2 × large diameter × (small diameter)^2^. For a model of lung metastasis in vivo, nude mice were injected with 100 μl WT (wide type), sh-FTO, ERBB2-OE and sh-FTO+ERBB2-OE KYSE150 stable cells (1 × 10^6^ cells per mouse, n = 3 for each group) through tail vein, respectively. Six weeks after injection, the mice were killed and analyzed for metastatic lung tumors. All animal research procedures are carried out under a program approved by the Animal Laboratory Center of University of Science and Technology of China.

### Immunohistochemistry assays (IHC)

Tissue arrays were constructed using 28 pairs of human ESCC and corresponding paracancerous tissues, as well as nude mouse specimens. Paraffin-embedded sections and immunohistochemical staining were performed on all specimens to detect the expression of FTO for human ESCC and paracancerous tissues, and the expression of Vimentin and E-cadherin, for nude mouse specimens. In short, the slides were incubated overnight with the appropriate antibody at 4 °C and the secondary antibody at room temperature for 2 h. Then, Subsequent steps were performed using the immunohistochemical kit (Beyotime, FD008) and peroxidase (Beyotime, P0202). The intensity of staining was the staining intensity was detected using the IMAGE-PRO PLUS 6.0 software (Media Cybernetics, Silver Spring, MD, USA). Specific steps: (1) finding and measuring the section of interest; (2) adjusting the optical density; (3) acquiring, converting and saving the image; (4) correcting the background and background staining; (5) configuring the section of interest to determine the light density; (6) Checking the optical density. The positive staining in images was quantified as integral optical density (IOD)/area, that is, mean density = density sum/area sum [36].The relevant clinical information was provided in Table S1.

### Bioinformatics analysis

Gene Expression Profiling Interactive Analysis (GEPIA) database was used to analyze the total expression level of FTO ESCC and normal esophageal epithelial samples. In addition, the correlation between YTHDF1 and ERBB2 expression was calculated. In addition, the protein–protein interaction among FTO and m^6^A writers was predicted using the STRING database.

### Vector and m^6^A mutation assays

Based on MeRIP-seq and mRNA-seq data, the 3′-terminal untranslated region (3′UTR) of potential m^6^A site ERBB2 containing wild and mutated m^6^A motif was cloned into vector pGL3 for luciferase reporter gene analysis. The sequences was provided in SI Appendix, Table S2.

### Statistical analysis

All values are expressed as mean ± SD from at least three independent trials. Statistical analysis was performed using GraphPad Prism 7 software. Two-tailed Student’s t-test, One-way ANOVA, and Two-way ANOVA were used to determine the statistical significance between the groups. Spearman analysis was used for the correlation between the two independent groups. The coefficient R > 0.3 indicates a moderate positive correlation. Survival curves were plotted using the Kaplan–Meier method with the log-rank test. p < 0.05 (*p < 0.05, **p < 0.01, ***p < 0.001, ****p < 0.0001) was considered statistically significant.

## Results

### FTO was significantly up-regulated in ESCC and esophageal carcinoma cells

**Fig. 1:**
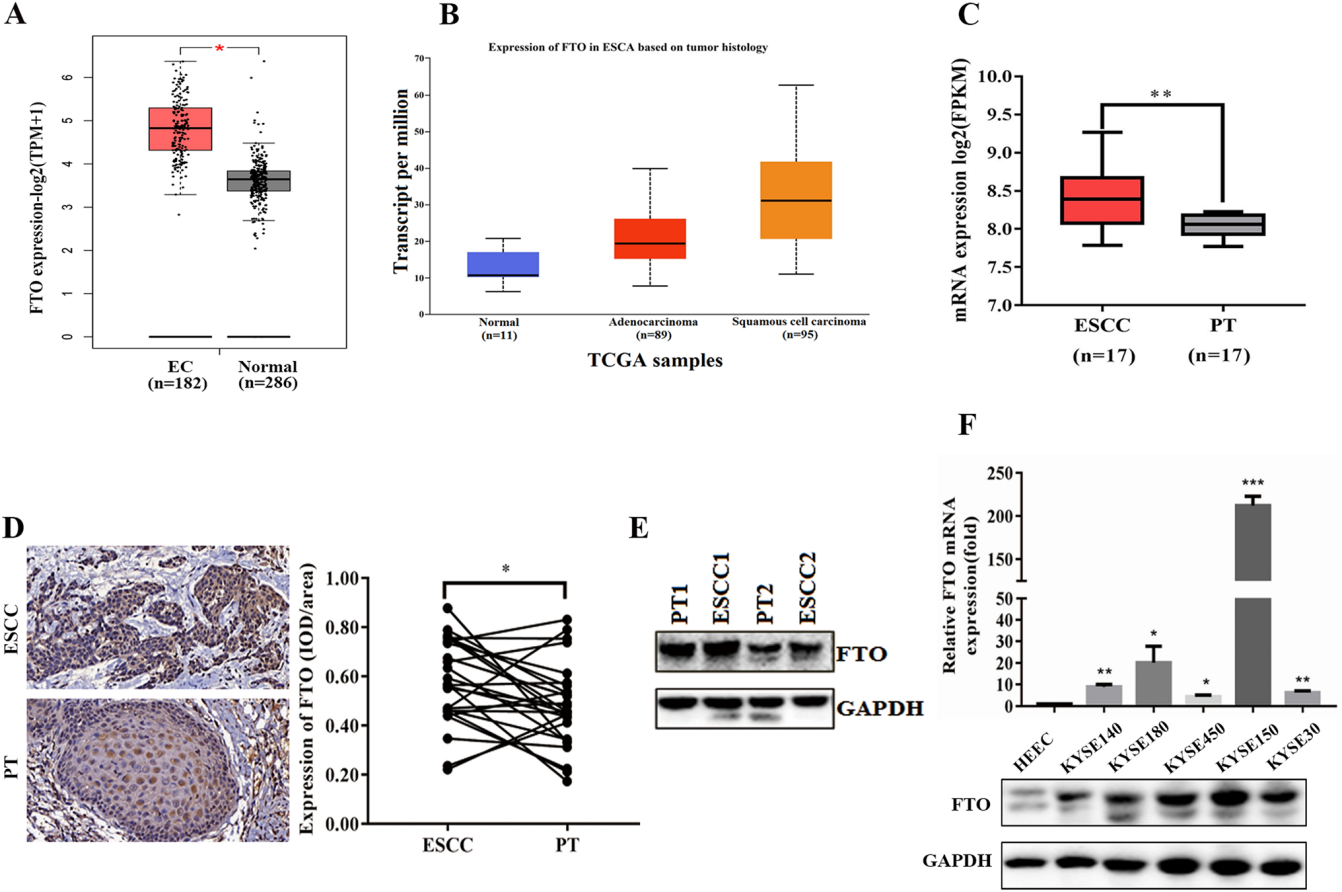
FTO was significantly up-regulated in ESCC and esophageal carcinoma cells. **A** FTO was significantly up-regulated in Esophageal carcinoma (EC) compared with normal Esophageal epithelial tissues from the GEPIA database data. **B** The expression levels (transcript per million) of FTO were analyzed in Esophageal adenocarcinoma (n = 89), Esophageal squamous cell carcinoma tissues (n = 95) and normal Esophageal epithelial tissues (n = 11) in the TCGA cohort. **C** The relative mRNA expression levels of FTO in 17 ESCC tissues and their adjacentnormal epithelial tissues (from the GSE20347 dataset) were analyzed. **D** Representative image of immunohistochemical staining by FTO in 400-fold magnified ESCC tissues and paired normal tissues in human samples (above).Immunohistochemical expression of FTO in ESCC tumor tissue and paired paracancerous tissues (n = 28) was quantitatively analyzed using IMAGE-PRO PLUS 6.0 software (below). **E** Western blotting assay of FTO expression in three paired in 2 pairs of ESCC primary tumor samples and their matched paracancerous tissues. **F** Real-time PCR (n = 3) and western blotting analysis of FTO expression in five ESCC cell lines and one normal esophageal cell line. Two-tailed Student’s t-test and one-way ANOVA were used to perform comparison between two groups and more groups, respectively. *p < 0.05,**p < 0.01; ***p < 0.001.

To explore the potential role of FTO in ESCC, we analyzed the expression level of FTO in ESCC patient tissues. Data from the GEPIA database showed that FTO was significantly upregulated in 182 esophageal cancer tissue samples compared to 286 normal esophageal epithelial tissue samples (Fig. 1A). To further identify whether FTO expression is associated with esophageal cancer histology, we analyzed The Cancer Genome Atlas (TCGA) data through the UALCAN database and found that FTO expression was significantly upregulated in 95 ESCC specimens compared to 11 normal esophageal epithelial cells. In addition, FTO expression was also up-regulated in 89 adenocarcinoma specimens, but without significant difference (Fig. 1B). In addition, ESCC has been reported to account for approximately 90% of esophageal cancers [37]. In the next step, FTO was also significantly upregulated in ESCC tissues compared to normal esophageal epithelium tissue based on GSE20347 dataset that microarray analysis of the gene expression profiles of 17 paired ESCC specimens and normal esophageal epithelium tissues (Fig. 1C).Consistently, the immunohistochemical (IHC) staining of the tissue arrays of 28 pairs of ESCC specimens and adjacent normal esophageal epithelial tissues also showed that the expression level of FTO was significantly higher than that of normal tissues (Fig. 1D). Furthermore, western blotting of FTO in two ESCC samples showed that FTO expression in primary tumor samples was significantly higher than that in the corresponding paracancerous tissues (Fig. 1E). Next, we examined FTO mRNA and protein levels in five ESCC cell lines, with the normal esophageal epithelial cell line (HEEC) as a control. The results showed that mRNA levels were significantly up-regulated in all five ESCC cells, with KYSE150 of the highest level (Fig. 1F). In addition, the protein levels were also greatly increased in each of the five ESCC cell compared to normal cells (HEECs) (Fig. 1F). All these results clearly indicated that FTO is highly expressed in ESCC patients, which may be a potential therapeutic biomarker for ESCC.

### FTO involves in the cell proliferation, migration and invasion of ESCC

**Fig. 2:**
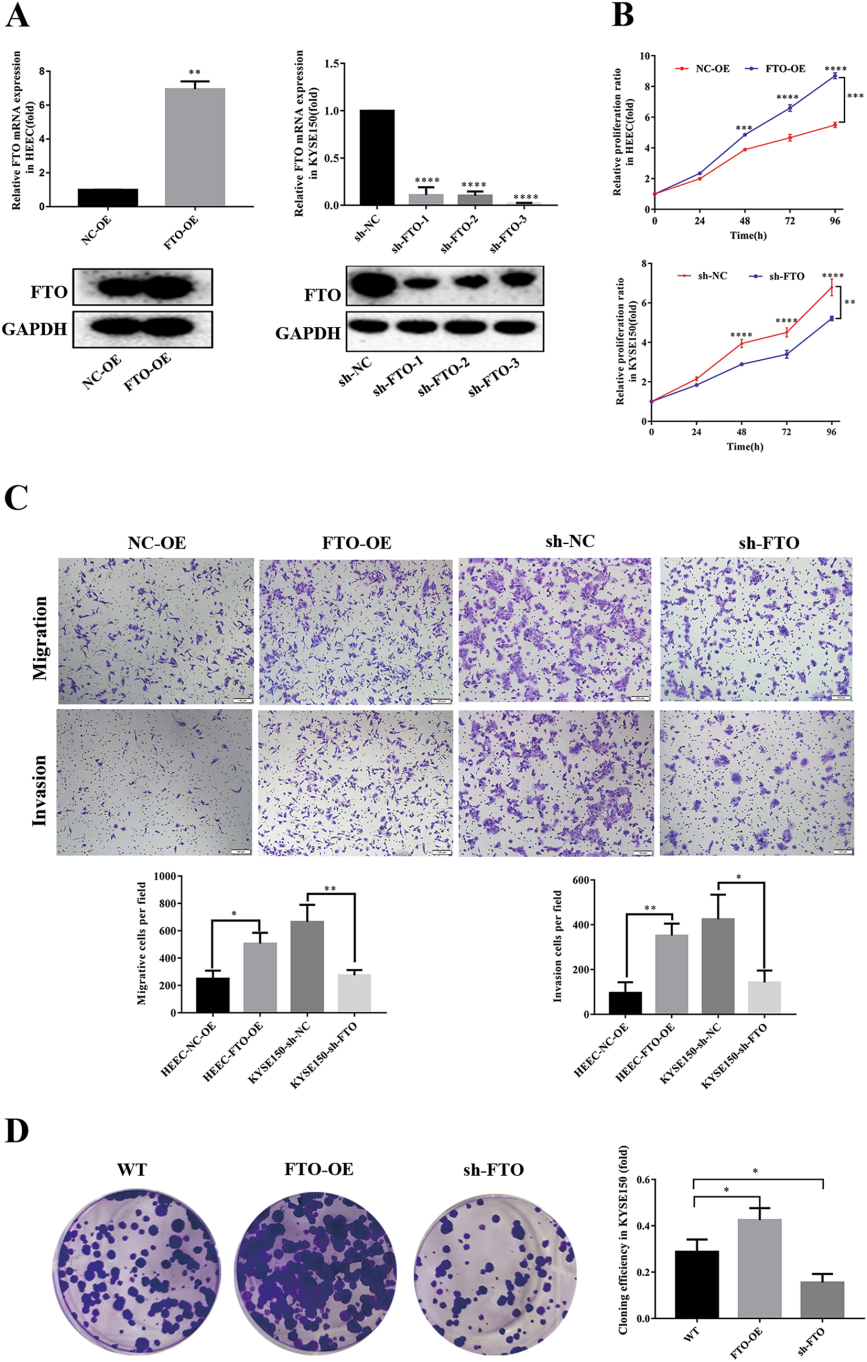
FTO regulates ESCC cell proliferation and migration, invasion and colony formation. **A** The over-expression efficiency of FTO was identified at RNA and protein levels using PCR (top) and western blotting (bottom). The interference efficiency of FTO was identified at RNA and protein levels by PCR (top) and western blotting (bottom). **B** CCK-8 assay was used to analyze the proliferation efficiency of cells after interfering with the expression efficiency of FTO. Overexpression of FTO (FTO-OE) significantly increased the proliferation efficiency of HEEC cells, while inhibition of FTO (sh-FTO) significantly decreased the proliferation efficiency of KYSE150 cells. **C** Transwell assay was used to assay the invasion and migration ability of HEEC cells. Overexpression of FTO (FTO-OE) significantly increased the invasion and migration ability of HEEC cells, while inhibition of FTO (sh-FTO) significantly decreased the invasion and migration ability of KYSE150 cells. **D** Colony formation assay was used to analyze the clone formation ability of KYSE150 cells after inhibition or overexpression of FTO. Results were presented as means ± SD (n = 3 per group). The two-tailed Student’s t-test, one-way ANOVA and two-way ANOVA were used to perform comparison between two groups and more groups, respectively. *p < 0.05, ***p < 0.001

To investigate the potential roles of FTO in ESCC, we performed a series of in vitro functional assays. First, we attempted to up-regulate or down-regulate FTO expression in KYSE150 cells. The qRT-PCR and western blotting analysis showed a significantly higher expression of FTO in both mRNA and protein levels by transfecting FTO-overexpressing lentivirus in HEEC cells (Fig. 2A). Moreover, transfection of each of the three different shRNAs of FTO in KYSE150 dramatically decreased the FTO expression in both mRNA and protein levels (Fig. 2A). The following CCK-8 assays in 96 h showed that HEEC cells with FTO overexpression possess a higher cell proliferation capability compared to the control cells, whereas silence of FTO expression significantly decreased the cell proliferation ratio in KYSE150 cells (Fig. 2B).

Afterwards, we performed the transwell assays to detect the invasion and migration capabilities upon up-regulating FTO in the normal HEEC cell or down-regulating FTO in the ESCC cell line KYSE150. The results showed that FTO overexpression in HEEC cells increased the cell migration and invasion capability compared to that of the control cells (Fig. 2C). In contrast, FTO down-regulation in KYSE150 cells inhibited the cell migration and invasion to a great extent (Fig. 2C).

Then, the colony formation assays were performed in KYSE150 cells to further compare the cell proliferation capability. As shown in Fig. 2D, more colonies were formed upon overexpression of FTO in KYSE150 cells, compared to the much less colonies formed in FTO-silenced KYSE150 cells (Fig. 2D). The results also confirmed that FTO overexpression promoted the ESCC cell proliferation. All these results indicated an oncogenic role of FTO, which involved in the cell proliferation, migration and invasion in ESCC.

### FTO regulates the level of m^6^A modification in ESCC cells

**Fig. 3:**
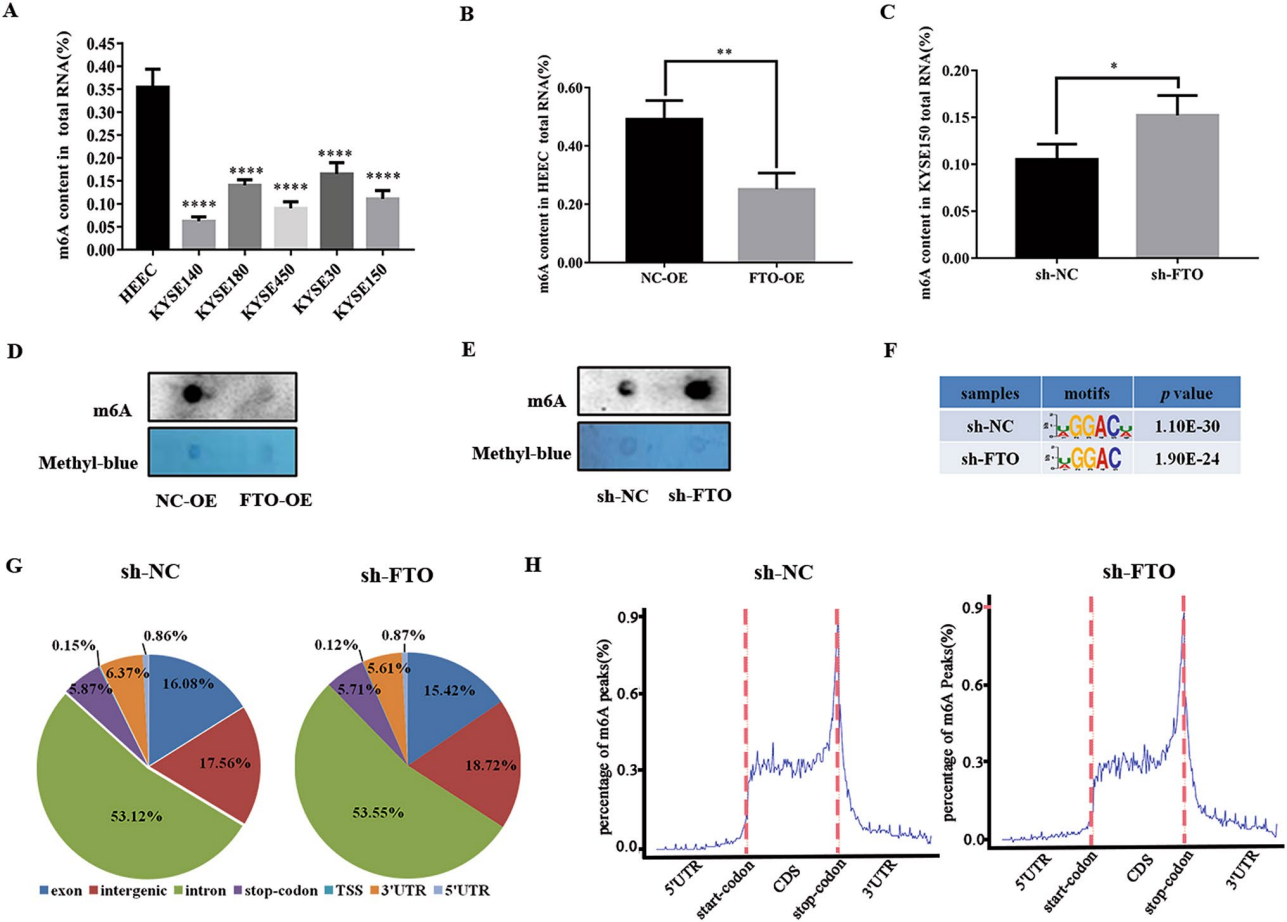
FTO regulates the level of m^6^A modification in ESCC cells. **A** The content of m^6^A in total mRNA in five ESCC cell lines and normal esophageal epithelial cell (HEEC) was determined by the m^6^A RNA methylation quantitative kit. **B**, **C** The RNA m^6^A content was determined with the m^6^A methylation kit after overexpression FTO (FTO-OE) in HEEC (**B**) and FTO-knockdown (sh-FTO) in KYSE150 cells (**C**). **D**, **E** Dot blot assay was used to analyze the methylation of m^6^A RNA after overexpression FTO (FTO-OE) in HEEC (**D**) and FTO-knockdown (sh-FTO) in KYSE150 cells (**E**). **F** Predominant consensus motif GGAC was detected in both the control and sh-FTO KYSE150 cells in m^6^A-seq. **G** Proportion of m^6^A peak distribution in the exon, intergenic, intron, stop-codon, transcription start site (TSS), 3′-untranslated region (3′UTR), 5′-untranslated region (5′UTR) across the entire set of mRNA transcripts. **H** Density distribution of m^6^A peaks across mRNA transcripts. Regions of the 5′UTR, coding region (CDS), and 3′UTR were split into 100 segments, the percentage of m^6^A peaks in each segment was determined. The data was presented as the mean ± SDs (n = 3). The two-tailed Student’s t-test and one-way ANOVA were used to perform comparison between two groups and more groups, respectively. *p < 0.05,**p < 0.01; ****p < 0.0001

As FTO is the well-investigated mRNA m^6^A demethylase, we therefore further analyze the m^6^A modification in five ESCC cell lines and one normal esophageal epithelial cell. The m^6^A RNA methylation kit was used to quantitatively measure the relative content of m^6^A modification. As expected, these cell lines with a higher FTO expression have a much lower content of RNA m^6^A modification, ranging from 5 to 20%, compared to the control cells (Fig. 3A). To further test the role of FTO on RNA m^6^A modification, we compared the m^6^A content with the control cells upon either FTO overexpression in HEEC cells or silencing in KYSE150 cells. As expected, upon the up-regulation of FTO in HEEC cells, the content of m^6^A modification among the total RNA greatly decreased from ~ 50 to ~ 30% (Fig. 3B). In contrast, down-regulation of FTO in KYSE150 cells led to an elevated level of m^6^A modification from ~ 10 to ~ 15% (Fig. 3C). In addition, the dot blot assays also showed a similar effect on the m^6^A content upon FTO overexpression in HEEC cells or FTO knockdown in KYSE150 cells (Fig. 3D, E). Sequence analysis of the m^6^A-seq data revealed the predominant consensus motif GGAC in KYSE150 cells (Fig. 3F). These results clearly showed that FTO indeed functions as a demethylase to remove mRNA m^6^A modification in ESCC cells.

Next, we analyzed the m^6^A peak distribution in the different regions of the mRNA transcripts in the KYSE150 cells. The results showed that the m^6^A peaks are mostly enriched in the intron (> 50%), followed with a large proportion present in the intergenic (~ 18%) and exon (~ 16%) regions (Fig. 3G). In contrast, there are only minor proportions of m^6^A peaks in the stop-codon, transcription start site (TSS), 3′-untranslated region (3′UTR), or 5′-untranslated region (5′UTR) (Fig. 3G). Notably, no significant difference was found in the KYSE150 cells with or without FTO knockdown, thus FTO did not alter the m^6^A distribution in the mRNA transcripts. We then analyzed the density distribution of m^6^A peaks across mRNA transcripts by splitting the corresponding regions into 100 segments. The data showed that the m^6^A modification mainly distributed in the coding region of mRNA transcripts, with the highest peak near by the stop-codon region (Fig. 3H).

### ERBB2 is the target of FTO in ESCC cells

**Fig. 4:**
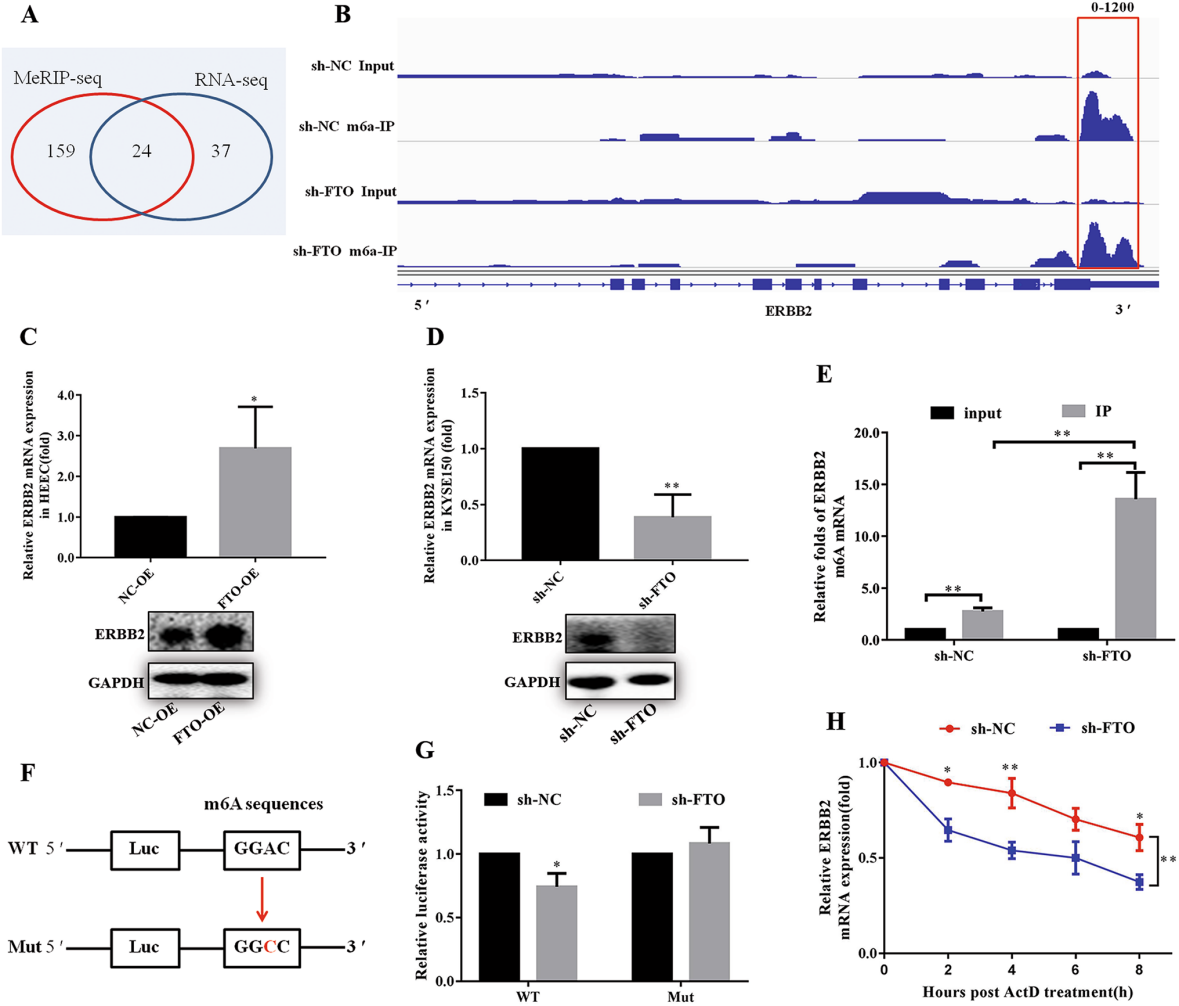
ERBB2 was identified as a downstream target of FTO. **A** Venn diagram showing identified differentially expressed genes using MeRIP-seq and RNA-seq in FTO stable knockdown KYSE150 cell compared with corresponding control. **B** m^6^A peaks were enriched in stop-codon and 3′UTRs of ERBB2 genes from m^6^A RIP-seq data. Squares marked increases of m^6^A peaks in ESCC cells with stable FTO down regulation compared with control cells. **C**, **D** The mRNA and protein levels of ERBB2 in FTO up-regulated cell (**C**) and down-regulated (**D**) cell were detected by q-PCR and western blot. **D** The mRNA and protein levels of ERBB2 in FTO down-regulated cells were detected by q-PCR and western blot. **E** m^6^A RIP-qPCR analysis of ERBB2 mRNA in the control and knockdown FTO cells. **F** Schematic representation that ERBB2 3′UTR wild (GGAC) and mutated (GGCC) were inserted into PGL vector. **G** Luciferase assay showed that knockdown of FTO repressed the expression of wide-type ERBB2 reporter. **H** The mRNA level of ERBB2 in KYSE150 cells with FTO knockdown and then treated with Actinomycete D (6 μg/ml) in 0, 2, 4, 6, and 8 h measured by real-time PCR. The data was presented as the mean ± SD (n = 3). The two-tailed Student’s t-test, one-way ANOVA and two-way ANOVA were used to perform comparison between two groups and more groups, respectively. *p < 0.05, **p < 0.01

To identify the molecular mechanism of FTO-regulated ESCC metastasis, we attempted to identify the potential targets of FTO in ESCC by analyzing the m^6^A contents in total mRNA using MeRIP-seq and RNA-seq in FTO-silenced KYSE150 cells and the control cells (Fig. 4A). The MeRIP-seq identified 183 genes (fold change > 2, p < 0.05) with m^6^A peak enriched in the stop codon and 3′UTR regions after FTO knockdown (Fig. 4A; Table S1). In addition, the RNA-seq data showed that totally 61 transcripts were markedly changed upon FTO knockdown (Fig. 4A; Table S2). Notably, 24 transcripts were shared in RNA-seq and MeRIP-seq data, in which 12 transcripts were up-regulated, whereas the other 12 transcripts were down-regulated (Fig. 4A).

Among the 12 shared down-regulated transcripts, we selected the top seven most differentially expressed members and detected their expression in KYSE150 cells by real-time PCR. The results showed that METTL1, CAMKK1, PKN2, and TGFBR1 were upregulated in the FTO-silenced KYSE150 cells, whereas ERBB2, AKT3 and PAK1P1 were downregulated. We selected the ERBB2 gene for further studies, which encodes the epidermal growth factor receptor that was previously found to be involved in the tumorigenesis of many different types of cancers [34, 38, 39]. The m^6^A RIP-seq data showed that the m^6^A peaks were enriched in the stop-codon and 3′UTRs of ERBB2 transcript in KYSE150 cells with or without FTO knockdown. Compared to the control, m^6^A methylation was up-regulated in the vicinity of the stop codon of ERBB2 after FTO knockdown (Fig. 4B).

To further investigate the correlation between ERBB2 and FTO, we detected the expression level of ERBB2 in FTO-overexpressed HEEC cells or FTO-silenced KYSE150 cells. The q-PCR and western blot analysis showed that accompanied with the up-regulation of FTO in HEEC cells, expression of ERBB2 is also significantly increased in both mRNA and protein levels (Fig. 4C). In contrast, silence of FTO in KYSE150 cells led to a dramatic decrease of ERBB2 in both mRNA and protein levels (Fig. 4D). The following m^6^A RIP-qPCR analysis showed that FTO down-regulation results in a higher content of m^6^A modification of ERBB2 transcript compared to the control cells. These results indicated that the demethylation of ERBB2 m^6^A might be catalyzed by FTO.

Therefore, we generated luciferase reporters containing a firefly luciferase and 3′UTR regions of wild-typeERBB2 or mutant (Fig. 4F). The results showed that knockdown of FTO in KYSE150 cells repressed the expression of wild-type ERBB2 reporter, whereas the mutant has a minor effect on ERBB2 expression (Fig. 4G). The results indicated that FTO demethylates the m^6^A modification of ERBB2 mRNA, which might affect its stability. To further prove this, we tested the ERBB2 mRNA levels in KYSE150 cells with FTO knockdown after the treatment with Actinomycete D, which is metabolic inhibitor [40, 41]. The results showed that the mRNA level is dramatically decreased along the time with FTO knockdown (Fig. 4H), indicating that FTO might increase the stability of ERBB2 mRNA, probably via regulating the m^6^A modification of ERBB2 mRNA.

### The inhibition effects of loss FTO are reversed by overexpression of ERBB2

**Fig. 5:**
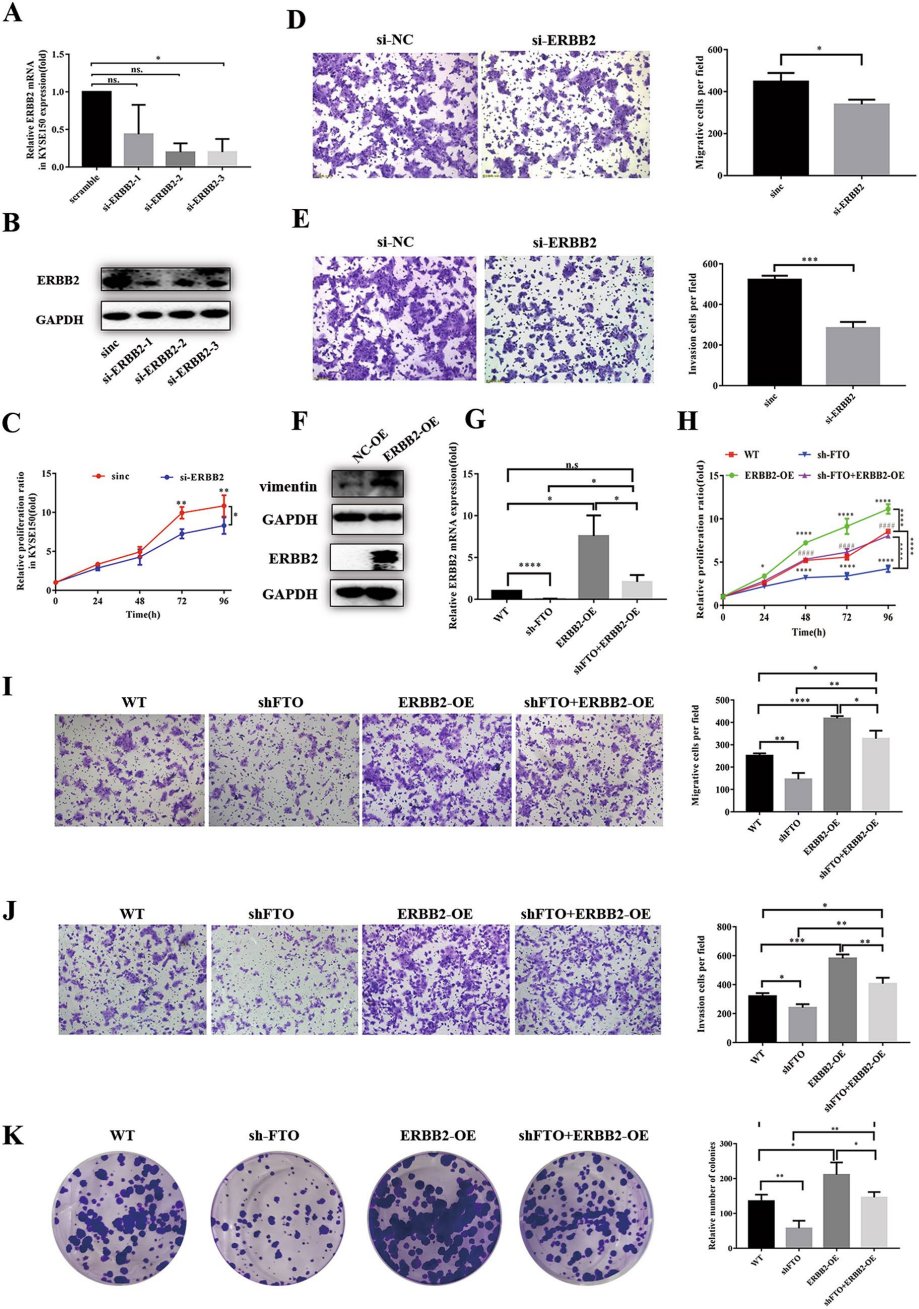
ERBB2 regulates the invasion and metastasis of ESCC cells and reverses biological effects of FTO. **A**, **B** RT-PCR and western blot was used to analyze the mRNA and protein level of ERBB2 in KYSE150 cells. **C** The CCK-8 assays showed that down-regulation of ERBB2 could inhibit the proliferation of KYSE150 cells. **D**, **E** Transwell assay was used to analyze the ability of down-regulation of ERBB2 to inhibit KYSE150 cell invasion and migration. The bar chart shows the corresponding quantitative analysis (right). **F** Western blot experiment verified the efficiency of the up-regulation ERBB2 expression and the promotion of EMT by the up-regulation ERBB2 expression. **G** The up-regulation of ERBB2 expression restored the down-regulation of FTO to the inhibition of ERBB2 expression using RT-PCR. **H** CCK-8 assay confirmed that up-regulation ERBB2 expression restored down-regulated FTO to KYSE150 cell proliferation inhibition. **I**, **K** and **H** Migration (**I**) invasion (**K**) and colony formation (**H**) assays showed that up-regulation of ERBB2 expression restored down-regulated FTO to KYSE150 cell migration, invasion and proliferation inhibition. The bar chart shows the corresponding quantitative analysis (right). The data was presented as the mean ± SD (n = 3). The two-tailed Student’s t-test, one-way ANOVA and two-way ANOVA were used to perform comparison between two groups and more groups, respectively. *n*.*s* no statistical significance. *p < 0.05, **p < 0.01, ***p < 0.001, ****p < 0.0001

We then characterized the roles of ERBB2 on ESCC cellular functions by several in vitro experiments. We first down-regulated the ERBB2 expression in KYSE150 cells via individually transfecting three ERBB2 siRNAs. The following qRT-PCR and western blot analysis showed that either of the three siRNAs could significantly inhibit the ERBB2 expression in both mRNA and protein levels (Fig. 5A, B). Accompanied with the decrease of ERBB2 level by si-ERBB2 transfection in KYSE150 cells, the cell proliferation ratio is significantly lower than that of the control cells (Fig. 5C). Moreover, the invasion and migration capabilities of ERBB2-slienced KYSE150 cells were largely decreased compared to the control cells (Fig. 5D, E). Notably, overexpression of ERBB2 significantly increased the expression of Vimentin (Fig. 5F), which is a major constituent of the intermediate filament family of proteins, and is known to maintain cellular integrity and provide resistance against stress. In addition, Vimentin is overexpressed in various epithelial cancers, including prostate cancer, gastrointestinal tumors, tumors of the central nervous system, breast cancer, malignant melanoma, and lung cancer [42–44], indicating that ERBB2 promotes ESCC cell migration. All these results indicated that ERBB2 involves in the tumorigenesis of ESCC progression.

To further investigate the correlation between FTO and ERBB2 on ESCC tumorigenesis, we overexpressed ERBB2 in KYSE150 cells accompanied with the slience of FTO. As shown in Fig. 5G, compared to the transfection of ERBB2-overexpressed vector (ERBB2-OE) that increased the ERBB2 mRNA expression to ~ tenfold, the ERBB2 expression in FTO-silenced KYSE150 cells with transfection of ERBB2-overexpressed vector (sh-FTO+ERBB2-OE) increased to ~ 2.5-fold, which largely compromises the effect of ERBB2 up-regulation. It also coincides with the notion that FTO-regulated m^6^A demethylation promotes the ERBB2 mRNA stability (Fig. 4H). As a result, the ERBB2 protein levels are up-regulated in the ERBB2-OE and sh-FTO+ERBB2-OE KYSE150 cells (Fig. 5G), but is much lower in the sh-FTO KYSE150 cells. To test whether ERBB2 could restore the effect of FTO knockdown, we tested the proliferation ratio of KYSE150 cells using CCK-8 assays. The results showed that FTO knockdown decreased the proliferation ratio, which could be restored by ERBB2 overexpression in the sh-FTO+ERBB2-OE cells (Fig. 5H). Similarly, the migration, invasion and colony formation assays showed that the effects of FTO knockdown in KYSE150 cells could also be restored by ERBB2 overexpression (Fig. 5I–K). These results suggested that FTO-regulated m^6^A demethylation on ERBB2 is associated with the tumorigenesis and metastasis in ESCC cells.

### YTHDF1 stabilizes ERBB2 mRNA via recognizing the m^6^A modification

**Fig. 6:**
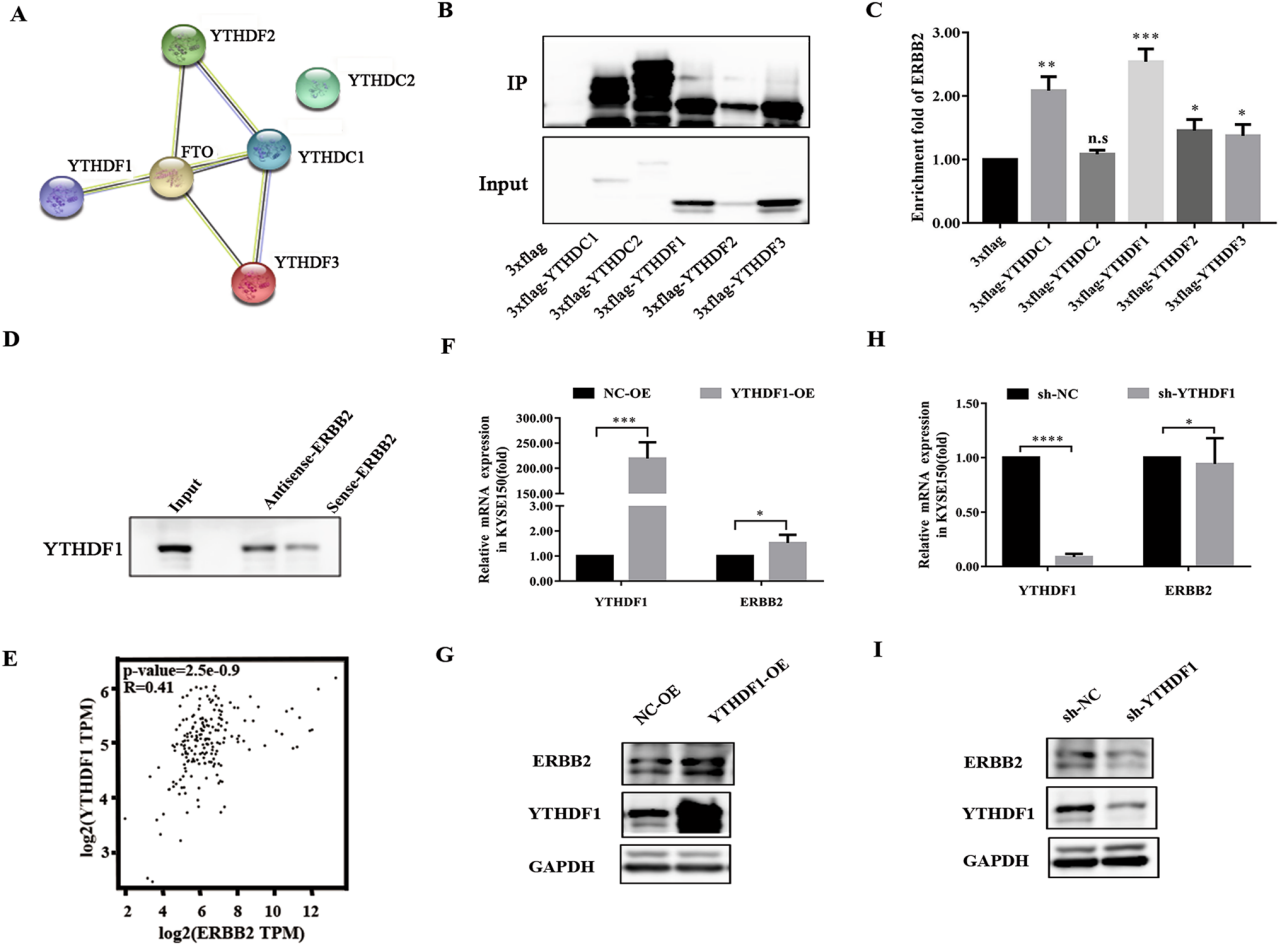
YTHDF1 preferentially decoded the m^6^A residue of ERBB2. **A** Protein–protein interaction network between FTO and m^6^A readers was analyzed using the STRING database. **B**, **C** Western blot (the left) and RIP-PCR (the right) were used to analyze the enrichment of 5 m^6^A recognition proteins in KYSE150 cells for ERBB2. The results showed that all proteins enriched the expression of ERBB2, however, ERBB2 was enriched to the greatest extent by YTHDF1. **D** The biotin-based pull down assays were used to further analyze the enrichment of YTHDF1 by ERBB2. The results showed a direct interaction between ERBB2 mRNA and YTHDF1. **E** The correlation between the expression of YTHDF1 and ERBB2 in ESCC was analyzed using GEPIA database and the results showed positive correlation. **F**, **G** The real-time PCR and the western blot analyzed showed that the expression of YTHDF1 and ERBB2 were detected in overexpression YTHDF1 (YTHDF1-OE) KYSE150 cells. **H**, **I** The real-time PCR and the western blot analyzed showed that the expression of YTHDF1 and ERBB2 were detected in knock-down YTHDF1 (sh-YTHDF1) KYSE150 cells. The result that The ERBB2 expression is related to YTHDF1 positively. The data in **C**, **F** and **H** was presented as the mean ± SD (n = 3). The two-tailed Student’s t-test and one-way ANOVA were used to perform comparison between two groups and more groups, respectively. *n*.*s* no statistical significance. *p < 0.05, **p < 0.01, ***p < 0.001, ****p < 0.0001

Previous studies had identified two major families of m^6^A “readers”, including the YTH family and the IGF2BP family, which might play roles in regulating the fate of methylated mRNA [45–47]. The STRING database indicated that FTO has potential interactions with the m^6^A readers of YTHDC1, YTHDF1, YTHDF2 and YTHDF3, but not YTHDC2 (Fig. 6A). To further identify the specific m^6^A readers of FTO, we performed the FLAG RNA pull-down assays in KYSE150 cells to screen the ERBB2-related m^6^A readers. Notably, ERBB2 was enriched by all members in the YTH family, with YTHDF1 of the greatest extent (Fig. 6B, C). Indeed, the biotin-based pull-down assays confirmed the direct interactions of ERBB2 mRNA with YTHDF1 (Fig. 6D). Furthermore, the bioinformatics analysis from the GEPIA database also indicated YTHDF1 negatively regulate the expression of ERBB2 (Fig. 6E).

To further test the role of YTHDF1 on ERBB2 expression and stability, we inhibited or increased the expression of YTHDF1 in KYSE150 cells, and detected the ERBB2 mRNA and protein levels. Accompanied with the up-regulated levels of YTHDF1 in KYSE150 cells, the ERBB2 mRNA and protein levels were significantly up-regulated (Fig. 6F, G). In contrast, ERBB2 mRNA and protein levels were somewhat down-regulated when YTHDF1 was silenced in KYSE150 cells (Fig. 6H, I). Taken together, our results suggested that the methylated ERBB2 transcripts might directly recognized by YTHDF1, which maintained the stability of the ERBB2 transcripts.

### FTO and ERBB2 promote in vivo ESCC progression

**Fig. 7:**
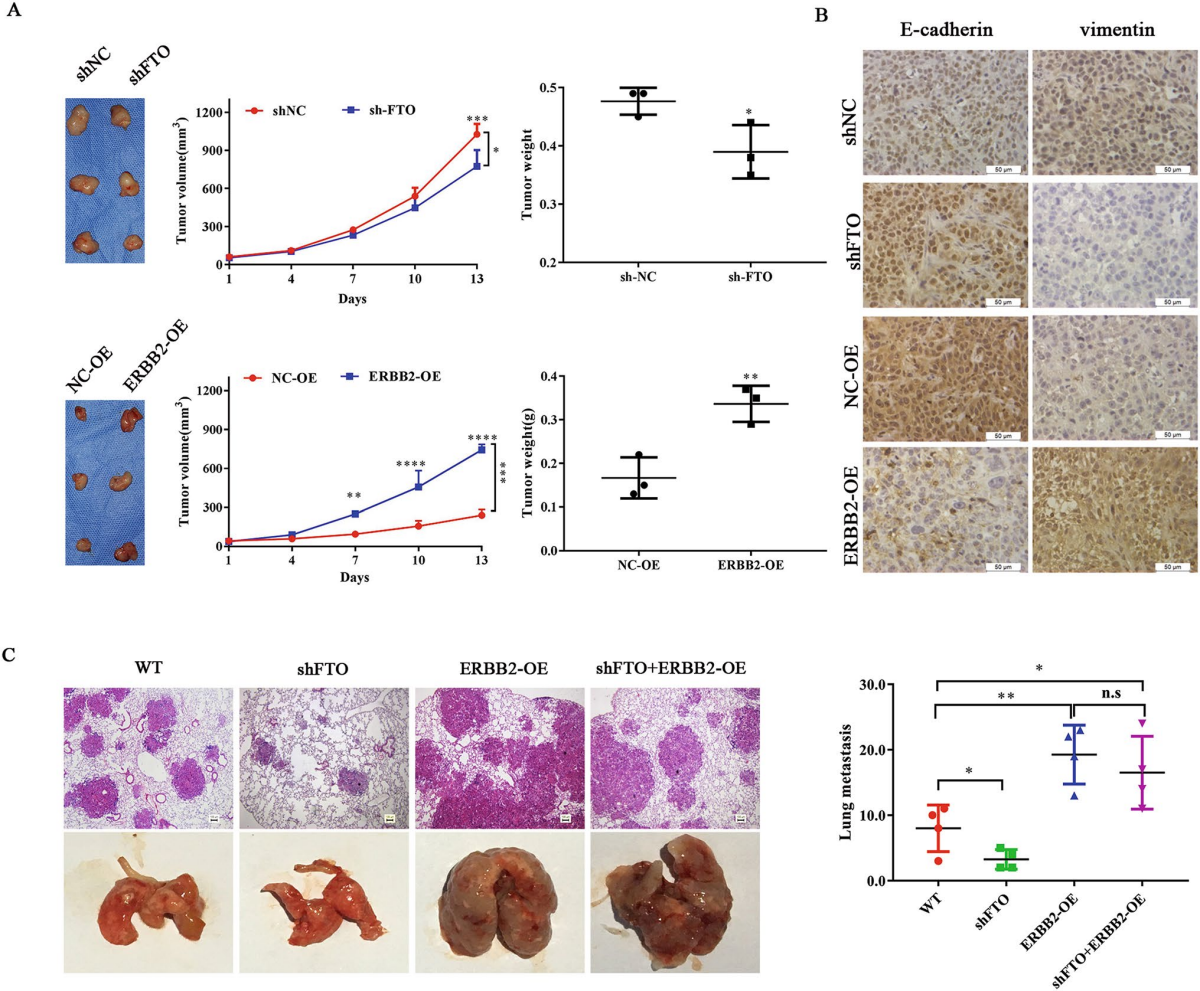
FTO and ERBB2 regulates in vivo ESCC progression. **A** The effects of FTO and ERBB2 on tumor formation, tumor weight and tumor volume change in nude mouse KYSE150-derived xenograft model. Representative images of tumors from FTO knockdown (sh-FTO) and up regulation expression ERBB2 (ERBB2-OE) versus the negative control sh-NC and NC-OE, respectively (n = 3 for each group).FTO knockdown effectively decreased ESCC subcutaneous tumor formation and growth. Overexpression of ERBB2 effectively increased ESCC subcutaneous tumor formation and growth. **B** The expression of Vimentin and E-cadherin, were analyzed from sh-NC, sh-FTO, NC-OE and ERBB2-OE KYSE150-derived xenograft by IHC. **C** Wide type (WT), FTO knockdown (sh-FTO), stable up regulation expression ERBB2 (ERBB2-OE) and FTO knockdown simultaneously stable up regulation expression ERBB2 (sh-FTO+ERBB2-OE) from KYSE150 cells were injected into the nude mice by tail vein injection. Representative images of metastatic lung tumors and the H&E staining results were shown (left), and the number of lung tumors was quantitatively analyzed (right). Results were presented as means ± SD (n = 4 per group). The two-tailed Student’s t-test and one-way ANOVA were used to compare the difference between two groups and more groups, respectively. *n*.*s* no statistical significance. *p < 0.05, **p < 0.01, ***p < 0.001, ****p < 0.0001

To test the potential role of FTO and ERBB2 on ESCC biogenesis in vivo, we injected the sh-FTO or ERBB2-OE KYSE150 cells subcutaneously into the nude mice. Compared to the control groups, FTO knockdown in KYSE150 cells effectively inhibited the tumor formation and growth (Fig. 7A). In contrast, overexpression of ERBB2 in KYSE150 cells enhanced the tumor growth (Fig. 7A). Further immunohistochemistry assays showed that FTO knockdown inhibited the expression of Vimentin and E-cadherin, indicating an inhibiting effect of tumor metastasis, whereas ERBB2 has a reverse effect (Fig. 7B). To further determine the impacts of m^6^A methylation on in vivo metastasis, sh-FTO, ERBB2-OE or sh-FTO&ERBB2-OE KYSE150 cells were injected into the nude mice, respectively. As shown in Fig. 7C, the lung metastasis was significantly inhibited upon injection of FTO silenced KYSE150 cells, accompanied with a decreased number of lung tumors. However, ERBB2-OE or sh-FTO & ERBB2-OE significantly promoted the lung metastasis and increased number of lung tumors compared with control cells (Fig. 7C). These results suggested that ERBB2 overexpression promoted tumor metastasis in vivo, which also confirmed the roles of FTO and ERBB2 involved in ESCC progression.

## Discussion

In this study, we uncovered a significant oncogenic role form^6^A modification in the tumorigenesis of ESCC. In brief, the decreased levels of mRNA m^6^A modification in ESCC is correlated with a higher level of FTO. A reversal change of FTO levels largely affects the in vitro proliferation, migration, and invasion of ESCC cells. Further investigations identified that ERBB2, an epidermal growth factor (EGF) receptor which belongs to subclass 1 of the receptor tyrosine kinase (RTK) superfamily [34, 48], is one of the targets of FTO. As found previously, ERBB2 was implicated in the development of human cancers [49]. For example, the ERBB2 alterations have been identified as oncogenic drivers and potential therapeutic targets in lung cancers [50, 51]. Here we showed that ERBB2 is also involved in the progression of ESCC, which provide the basis for further targeting ERBB2 pathway for clinical therapy of ESCC.

To date, the knowledge on the roles of mRNA modification in regulating cancer progression remains limited. As the first characterized m^6^A demethylase, FTO has been reported to regulate the tumorigenesis in different types of cancers. FTO was found to enhance leukemic oncogene-mediated cell transformation and leukemogenesis via reducing the m^6^A levels of its targets [33]. In addition, pharmaceutical inhibition of FTO by a chemical inhibitor suppresses tumor progression and substantially prolongs the lifespan of glioblastoma stem cell-grafted mice [52]. In this study, we provided the direct evidence of FTO in regulating the m^6^A methylation of ERBB2 mRNA. We found that FTO regulates multiple aspects of ESCC progression, including migration, invasion, proliferation, and tumorigenesis. Our results emphasized the roles of FTO-regulated m^6^A modification in cancer progression, and also provided hints to develop therapeutic strategies against ESCC metastasis by targeting m^6^A modification and its related targets.

The m^6^A modification modulates almost all stages in the life cycle of RNA, such as RNA processing, nuclear export, and translation modulation [20, 53]. Previous studies showed that the reader protein YTHDF1 associates with the progression of various cancers, including non-small cell lung cancer [54, 55], and ovarian cancer [56]. In our study, we found that knockdown of YTHDF1 decreased the ERBB2 expression in both mRNA and protein levels (Fig. 6), whereas YTHDF1 overexpression significantly enhanced the ERBB2 mRNA and protein levels. These data support the notion that YTHDF1 directly targets ERBB2, which enhanced the stability of ERBB2 mRNA transcripts. The results also indicated that YTHDF1 might regulate the transcription and translation of ERBB2. However, more investigations are needed to elucidate the fine mechanism of YTHDF1-regulated ERBB2 functions.

## Supplementary Information

Below is the link to the electronic supplementary material.

Supplementary file1 (PDF 631 KB)

## Data Availability

Not applicable.

## Abbreviations

ESCC: Esophageal squamous cell carcinoma
m^6^A: N^6^-methyladenosine
MeRIP-seq: Methylated RNA immunoprecipitation sequencing
RNA-seq: Transcriptomic RNA sequencing
FTO: Fat-mass and obesity-associated protein
ERBB2: Erb-B2 receptor tyrosine kinase 2
YTHDF1: YTH N^6^-methyladenosine RNA binding protein 1
YTHDF2: YTH N^6^-methyladenosine RNA binding protein 2
IGF2BP1: Insulin like growth factor 2 mRNA binding protein 1
IGF2BP2: Insulin like growth factor 2 mRNA binding protein 2
ActD: Actinomycetes D
E-cad: E-cadherin
VIM: Vimentin
WT: Wide-type
IHC: Immunohistochemical
RT-qPCR: Quantitative real-time PCR
RIP: RNA Immunoprecipitation
SDS-PAGE: Sodium dodecyl sulfate–polyacrylamide gel electrophoresis
3′UTR: Three prime untranslated region
CDS: Coding sequence
TCGA: The Cancer Genome Atlas
