# Supplementary material for: FTO mediated ERBB2 demethylation promotes tumor progression in esophageal squamous cell carcinoma cells
Source: Clin Exp Metastasis. 2022 May 7;39(4):623–39. doi: 10.1007/s10585-022-10169-4 (PMC9338917; doi:10.1007/s10585-022-10169-4)
Supplement: Supplementary file 1 — Supplementary file1 (PDF 631 KB) [file 10585_2022_10169_MOESM1_ESM.pdf]

Supplementary Information for

FTO mediated ERBB2 demethylation promotes tumor progression in esophageal squamous cell carcinoma cells

Supplementary Table S1.

The clinical and pathological information of ESCC patients

case	Gender	Age	grade	tumor type
NO.1	Male	66	II -III	invasive squamous cell carcinoma of esophagus
NO.2	Male	70	II	esophageal squamous cell carcinoma
NO.3	Male	72	II -III	invasive squamous cell carcinoma of esophagus
NO.4	Male	79	I	esophageal squamous cell carcinoma
NO.5	Male	76	III	invasive squamous cell carcinoma of esophagus
NO.6	Male	62	III	esophageal squamous cell carcinoma
NO.7	Male	66	II -III	esophageal squamous cell carcinoma
NO.8	Male	54	II -III	invasive squamous cell carcinoma of esophagus
NO.9	Male	80	II -III	invasive squamous cell carcinoma of esophagus
NO.10	Famale	63	III	invasive squamous cell carcinoma of esophagus
NO.11	Famale	72	II	esophageal squamous cell carcinoma
NO.12	Male	74	III	esophageal squamous cell carcinoma
NO.13	Male	88	II	esophageal squamous cell carcinoma
NO.14	Male	68	III	invasive squamous cell carcinoma of esophagus
NO.15	Male	88	II	esophageal squamous cell carcinoma
NO.16	Male	68	II -III	esophageal squamous cell carcinoma
NO.17	Famale	72	II	esophageal squamous cell carcinoma
NO.18	Male	52	II	esophageal squamous cell carcinoma
NO.19	Male	66	II	esophageal squamous cell carcinoma
NO.20	Male	67	I	esophageal squamous cell carcinoma
NO.21	Male	71	III	esophageal squamous cell carcinoma
NO.22	Male	75	II	esophageal squamous cell carcinoma
NO.23	Male	68	I	esophageal squamous cell carcinoma
NO.24	Male	64	II	esophageal squamous cell carcinoma
NO.25	Famale	73	III	esophageal squamous cell carcinoma
NO.26	Male	74	II -III	basal-like squamous cell carcinoma of the esophagus
NO.27	Male	66	I	invasive squamous cell carcinoma of esophagus
NO.28	Famale	65	II	invasive squamous cell carcinoma of esophagus

Supplementary Table S2

Information of sequences used in this study

Group	Names	Sequences
qRT PCR primer	hFTO-F	5'-GGAACAAAGGAATGAAATCTTGAC-3'
	hFTO-R	5'-TAATGTTCGGGCAATTCGTG-3'
	hFTO-Probe	5'-FAM-CCTTGCCTCGCTCACTGCACG-3'BHQ1
	hERBB2-F	5'-GACTGCTGCAGGAAACGGAG-3'
	hERBB2-R	5'-TGCCAAAAGCGCCAGATC-3'
	hERBB2-Probe	5'-FAM-CGGAGCGATGCCCAACCAGG-3'BHQ1
	hACTB-F	5'-GCCCATCTAXGAGGGGTATG-3'
	hACTB-R	5'-GAGGTAGTCAGTCAGGTCCCG-3'
	hACTB-Probe	5'-CY5-CCCCCATGCCATCCTGCGTC-3'BHQ1

	hYTHDF1-F	5'-GTGGACACCCAGAGAACAAA-3'
	hYTHDF1-R	5'-CAGTAAGGTAGGGCTCAAAGTC-3'
m^6A-qPCR primer	ERBB2-m^6A-F	5'-CTACGGCAGAGAACCCAGAG-3'
	ERBB2-m^6A-R	5'-TTGATGCCAGCAGAAGTCAG-3'
PCR primer for RNA pull-down	hERBB2-F	5'-ACCTGCTGAACTGGTGTATG-3'
	hERBB2-R	5'-TGACATGGTTGGGACTCTTG-3'
Biotin-Labeled Probe for RNA pull-down	sense-ERBB2	5'-biotinACCTGCTGAACTGGTGTATG-3'
	antisense-ERBB2	5'-biotinCATACACCAGTTCAGCAGGT-3'
Knockdown FTO Sequences	Negative control-F	5'-GATCCGTTCTCCGAACGTGTCACGTAATTCAAGAGATTACGTGACACGTTCGGAGAATTTTTTC-3'
	Negative control-R	5'-AATTGAAAAAATTCTCCGAACGTGTCACGTAATCTCTTGAATTACGTGACACGTTCGGAGAACG-3'
	sh-FTO-1-F	5'-GATCCGGATGACTCTCATCTCGAAGGTTCAAGAGACCTTCGAGATGAGAGTCATCCTTTTTTG-3'
	sh-FTO-1-R	5'-AATTCAAAAAAGGATGACTCTCATCTCGAAGGTCTCTTGAACCTTCGAGATGAGAGTCATCCG-3'
	sh-FTO-2-F	5'-GATCCGAAATAGCCGCTGCTTGTGAGATTCAAGAGATCTCACAAGCAGCGGCTATTTTTTTTTG-3'
	sh-FTO-2-R	5'-AATTCAAAAAAAAATAGCCGCTGCTTGTGAGATCTCTTGAATCTCACAAGCAGCGGCTATTTCG-3'
	sh-FTO-3-F	5'-GATCCGAAGAGCAGAGCAGCATACAACGTAATTCAAGAGATTACGTTGTATGCTGCTCTGCTCTTTTTTTTG-3'
	sh-FTO-3-R	5'-AATTCAAAAAAAAGAGCAGAGCAGCATACAACGTAATCTCTTGAATTACGTTGTATGCTGCTCTGCTCTTCG-3'
Knockdown ERBB2 Sequences	Negative control-F	5'- UUCUCCGAACGUGUCACGUTT-3'
	Negative control-R	5'-ACGUGACACGUUCGGAGAATT-3'
	si-ERBB2-1-F	5'-GCUCUUUGAGGACAACUAUTT-3'
	si-ERBB2-1-R	5'-AUAGUUGUCCUCAAAGAGCTT-3'
	si-ERBB2-2-F	5'-GCAGUUACCAGUGCCAAUATT-3'
	si-ERBB2-2-R	5'-UAUUGGCACUGGUAACUGCTT-3'
	si-ERBB2-3-F	5'-GGUGUAUGCAGAUUGCCAATT-3'
	si-ERBB2-3-R	5'-UUGGCAAUCUGXAUACACCTT-3'
Knockdown YTHDF1 Sequences	sh-YTHDF1-F	5'-CCGGGGATACAGTTCATGACAATGAGGATCCTCATTGTCATGAACTGTATCCTTTTTG-3'
	sh-YTHDF1-R	5'-AATTCAAAAAGGATACAGTTCATGACAATGAGGATCCTCATTGTCATGAACTGTATCC-3'

Supplementary Table S3

12 up-regulated and 12 down-regulated transcripts were shared in RNA-Seq and MeRIP-Seq.

Group	Gene
up regulation	METTL1
	CAMKK1
	PKN2
	TGFBR1
	CAMKK2
	FGF2
	CDK6
	MET
	JAK1
	MAPK11
	MAP2K3
	SMAD3
down regulation	HMGCR
	MMP14
	ELK4
	IGF1R
	VDR
	ZFHX3
	FOS
	RELA
	PAK1
	ERBB2
	AKT3
	PAK1IP1

Supplementary Figures:

Fig. S1.

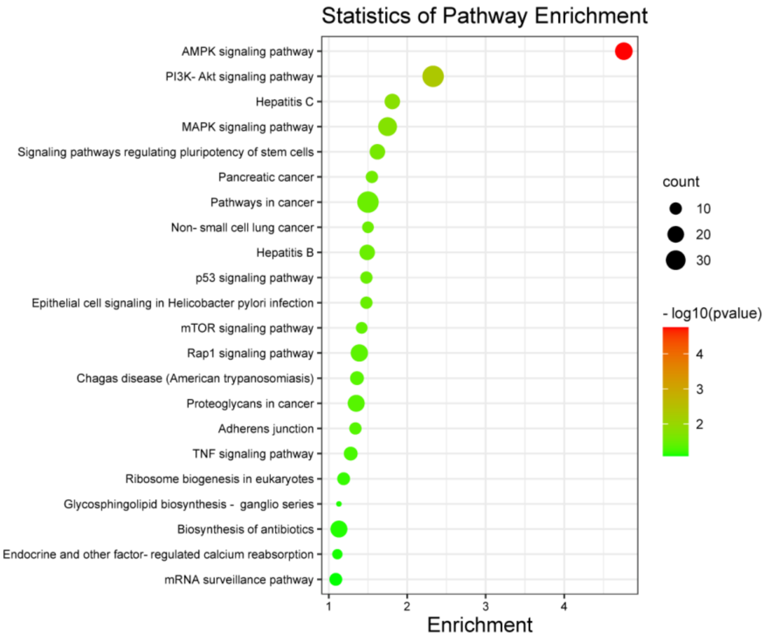

A cluster profiler identified the enriched KEGG processes of m^{6}A expression upregulation in ESCCs cells compared with control cells.

Fig. S2.

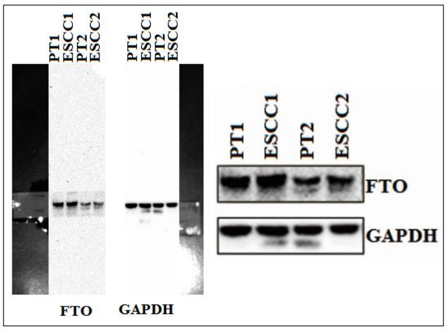

The full-length gels of the Figure1C, western analyses used in the manuscript.

Fig. S3.

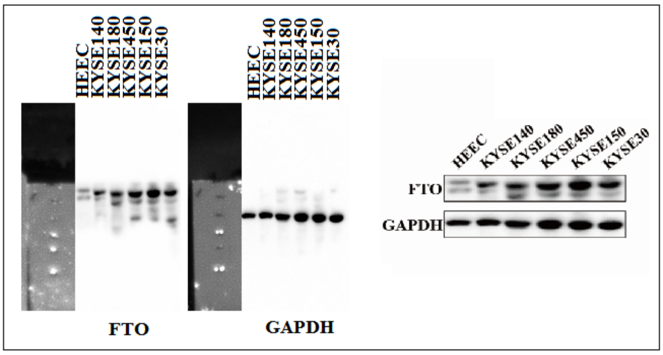

The full-length gels of the Figure1D, western analyses used in the manuscript.

Fig. S4.

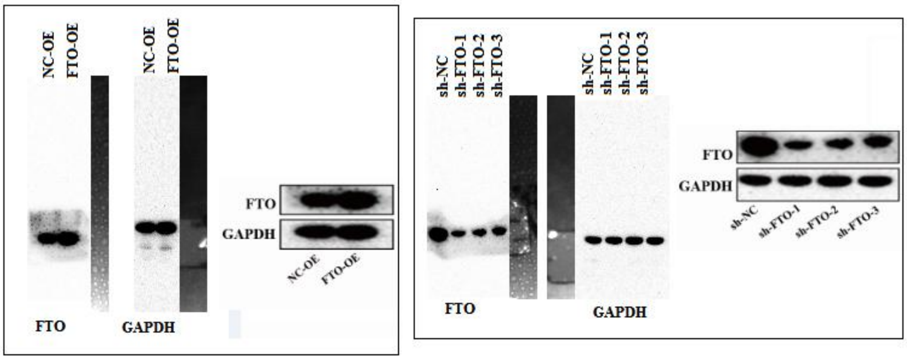

The full-length gels of the Figure2A, western analyses used in the manuscript.

Fig. S5.

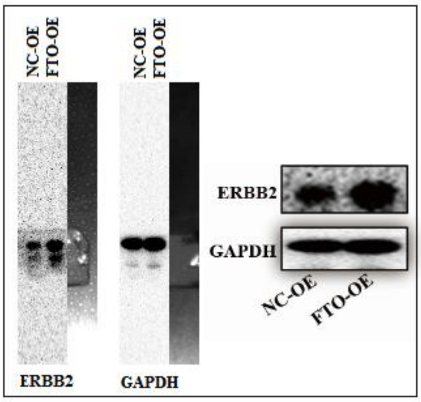

The full-length gels of the Figure4C, western analyses used in the manuscript.

Fig. S6.

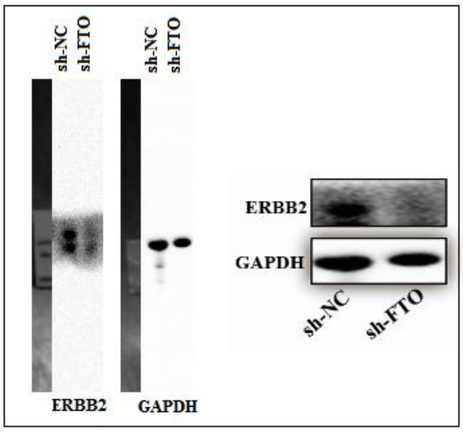

The full-length gels of the Figure4D, western analyses used in the manuscript.

Fig. S7.

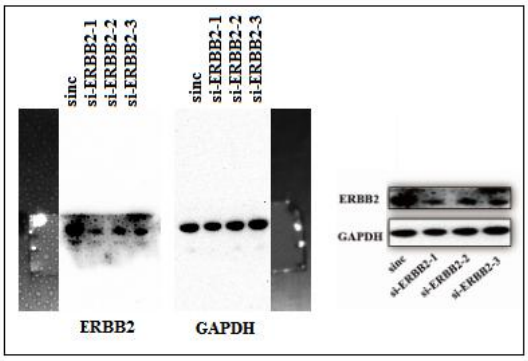

The full-length gels of the Figure5B, western analyses used in the manuscript.

Fig. S8.

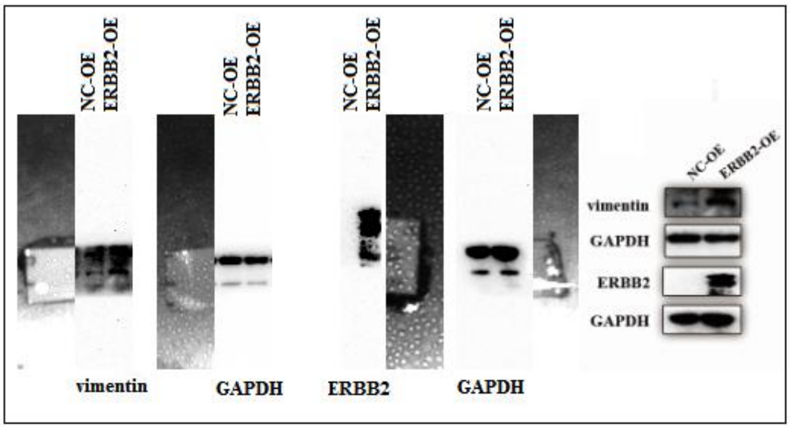

The full-length gels of the Figure5F, western analyses used in the manuscript.

Fig. S9.

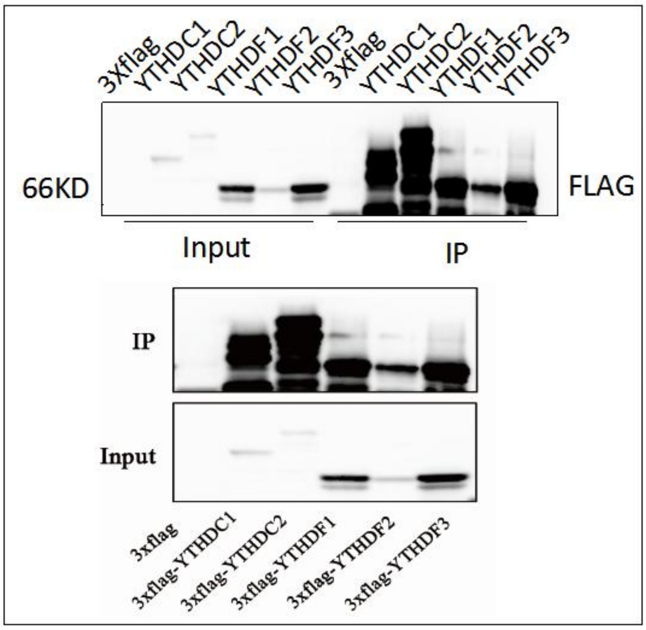

The full-length gels of the Figure6B, western analyses used in the manuscript.

Fig. S10.

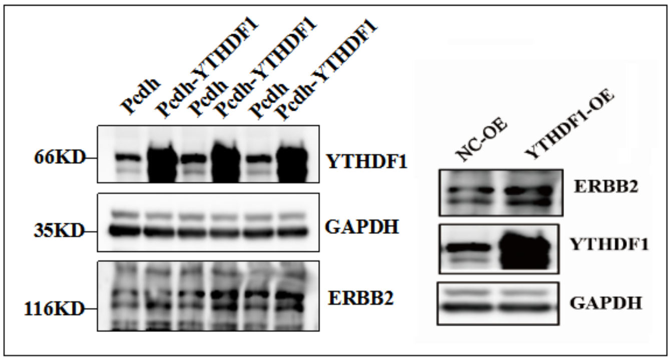

The full-length gels of the Figure6G, western analyses used in the manuscript.

Fig. S11.

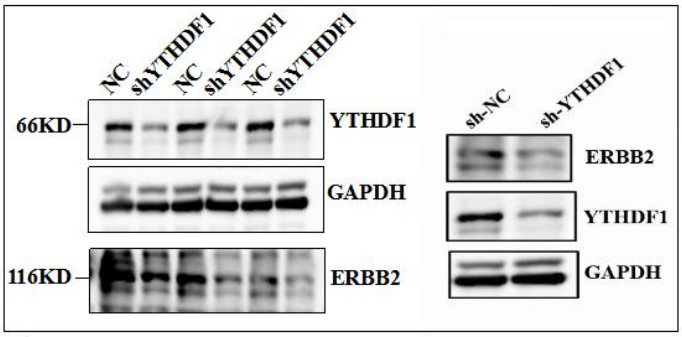

The full-length gels of the Figure6I, western analyses used in the manuscript.

Fig. S12.

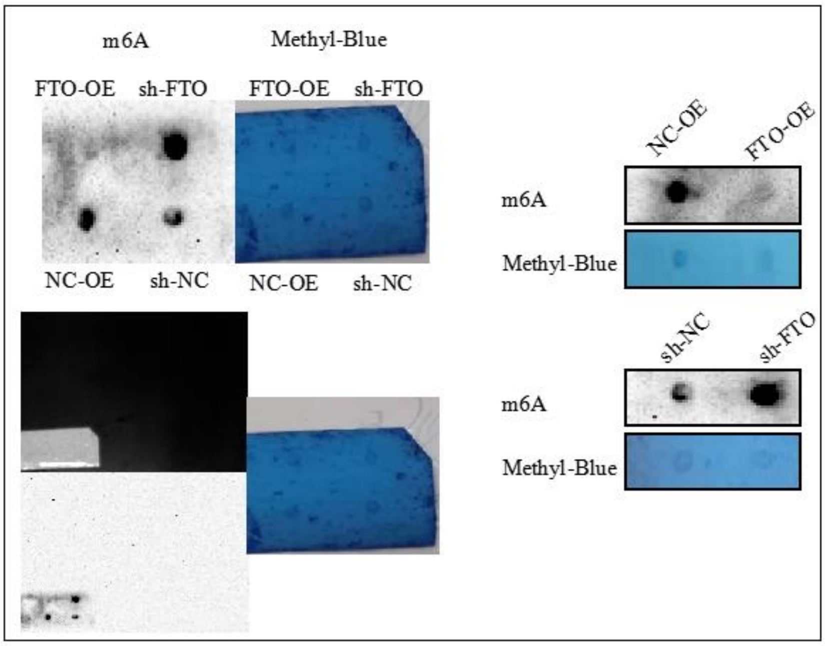

The full-length gels of the Figure3D and 3E, dot blot assays used in the manuscript.
